# Evaluating the Impact of gRNA SNPs in CasRx Activity for Reducing Viral RNA in HCoV-OC43

**DOI:** 10.3390/cells11121859

**Published:** 2022-06-07

**Authors:** Cathryn Michelle Mayes, Joshua Santarpia

**Affiliations:** 1 Sandia National Laboratories, Albuquerque, NM 87123, USA; 2 Department of Pathology and Microbiology, University of Nebraska Medical Center, Omaha, NE 68198, USA; josh.santarpia@unmc.edu

**Keywords:** CRISPR, CasRx, off-target effects, human coronaviruses, HCoV-OC43, homology

## Abstract

Viruses within a given family often share common essential genes that are highly conserved due to their critical role for the virus’s replication and survival. In this work, we developed a proof-of-concept for a pan-coronavirus CRISPR effector system by designing CRISPR targets that are cross-reactive among essential genes of different human coronaviruses (HCoV). We designed CRISPR targets for both the RNA-dependent RNA polymerase (RdRp) gene as well as the nucleocapsid (N) gene in coronaviruses. Using sequencing alignment, we determined the most highly conserved regions of these genes to design guide RNA (gRNA) sequences. In regions that were not completely homologous among HCoV species, we introduced mismatches into the gRNA sequence and tested the efficacy of CasRx, a Cas13d type CRISPR effector, using reverse transcription quantitative polymerase chain reaction (RT-qPCR) in HCoV-OC43. We evaluated the effect that mismatches in gRNA sequences has on the cleavage activity of CasRx and found that this CRISPR effector can tolerate up to three mismatches while still maintaining its nuclease activity in HCoV-OC43 viral RNA. This work highlights the need to evaluate off-target effects of CasRx with gRNAs containing up to three mismatches in order to design safe and effective CRISPR experiments.

## 1. Introduction

Human coronaviruses (HCoV) have a significant impact on public safety and human health, as underscored by the ongoing COVID-19 pandemic. In addition to SARS-CoV-2, there are two additional highly pathogenic HCoVs, which include severe acute respiratory syndrome coronavirus (SARS-CoV) and Middle East respiratory syndrome coronavirus (MERS-CoV). These viruses are known for their ability to cause global outbreaks due to their high pathogenicity and transmissibility. Moreover, on 5 October 2012, the National Select Agent Registry Program published a final rule declaring SARS-CoV a select agent because of its potential to pose a severe threat to public health and safety [1]. There are also four common HCoVs that cause mild to moderate upper-respiratory tract illnesses and contribute to 15–30% of common colds in adults. These include HCoV-229E, HCoV-OC43, HCoV-NL63, and HCoV-HKU1 [2]. While the disease severity associated with infections from these viruses is less than that of the highly pathogenic HCoVs, they still contribute to the burden on public health posed by coronaviruses. 

In order to address the threat of coronaviruses, we have developed a CRISPR effector system with the potential to cleave essential genes among different HCoV species. We utilized CasRx, which is a Cas13d type CRISPR effector derived from *Ruminococcus flavefaciens* strain XPD3002 [3]. It is an RNA-targeting endonuclease with specific cleavage that is directed by a guide RNA (gRNA) composed of a 30-nucleotide (n.t.) direct repeat (DR) plus a 22 n.t. spacer complementary to the targeted region. Cas13 effectors do not require a promoter flanking sequence in the targeted region, allowing for greater flexibility in which target sequence can be selected. Additionally, CasRx has proven to be highly effective in mammalian cells, with >90% knockdown of RNA in mammalian cells [3,4].

Because coronaviruses have an ssRNA genome, their mutation rate is much higher than that of DNA viruses, resulting in less genomic conservation among different HCoV species [5]. To account for this, we introduced up to three mismatches in the gRNA sequences to determine how effective the CasRx endonuclease is when there is sequence variability between the gRNA and target sequences. Previous work has analyzed the effect that gRNA mismatches have on Cas13 efficacy both computationally [6] and experimentally in organisms such as *Drosophila* [7] and human and mouse cells [8]. This work has found that Cas13 effectors may be tolerant to certain nucleotide mismatches [9,10], especially when present in the central region of the gRNA [6,7].

While others have previously evaluated the effects of mismatches on other Cas13 effectors, extensive evaluation of off-target effects of CasRx has not yet been conducted, so this work will provide insight into designing safe CRISPR experiments with this specific Cas13 effector. 

As a proof-of-concept study, we designed CRISPR targets that are cross reactive among essential genes of different human coronaviruses to develop a pan-coronavirus CRISPR effector system. We evaluated the effect of mismatches in gRNAs on the efficacy of CasRx binding and cleavage in regions of essential genes that are not perfectly homologous among different HCoV species. This work provides valuable insight into off-target effects of CasRx, which is critical for safely designing CRISPR experiments. Additionally, characterizing the effect of polymorphisms is important in considering the functionality of the CRISPR system as new variants develop and novel diseases arise.

## 2. Materials and Methods

### 2.1. HCoV-OC43 Propagation

HCoV-OC43 virus (ATCC, Catalog #VR-1558) was propagated in HCT-8 cells (ATCC, Catalog #CCL-244) when the host cells grew to 80–90% confluence. After aspirating media from the host cells, 2.5 mL of virus in RPMI medium was added to a T-75 cm^2^ flask at a multiplicity of infection (MOI) of 0.1. Infected cells were incubated at 33 °C, 5% CO_2_ for 1–2 h with continuous rocking, then 10 mL RPMI + 2% horse serum was added to the flasks and incubation continued for 4 days until cytopathic effects (CPE) developed. CPE included cell vacuolization and cell sloughing. The virus was harvested by scraping the cells into the medium and quick-freezing in liquid nitrogen vapor. The viral titer was determined by performing a median tissue culture infectious dose (TCID_50_) assay [11].

### 2.2. Designing Guide RNA (gRNA) Sequences Targeting Human Coronaviruses

**Figure 1:**
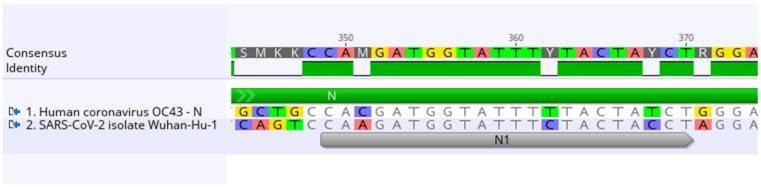
Comparing HCoV-OC43 (GenBank: AY585228) and SARS-CoV-2 (GenBank: NC_045512.2) CRISPR target sequences. Mismatches between HCoV-OC43 and SARS-CoV-2 sequences are highlighted based on nucleotide.

Similar to previous work, we performed sequencing alignment of individual HCoV-OC43 and SARS-CoV-2 genes using Geneious Prime 2021.1.1 software (Auckland, New Zealand, https://www.geneious.com, accessed on 17 March 2020) to identify essential genes with the highest level of conservation and homology [12]. We then designed gRNA within the conserved genes by targeting 22 n.t. homologous regions of the genes. When perfect homology between the sequences was not present, single nucleotide polymorphisms (SNPs) were introduced into the gRNA sequence. To design these targets with mismatches, nucleotides in the HCoV-OC43 target were substituted for those present in SARS-CoV-2 (Figure 1). Up to three mismatches were introduced into one gRNA, and the location of SNPs were labelled as A, B, and C for each gRNA. These SNPs were tested individually, in pairs, and with all three for each CRISPR target. We designed an additional gRNA that is a perfect match between SARS-CoV-2 and HCoV-OC43. We named this gRNA sequence RdRp_ctrl since it is a control for exact homology between both viruses.

### 2.3. Cloning Guide RNA (gRNA) Sequences Compatible with CasRx into Backbone Plasmid

**Figure 2:**
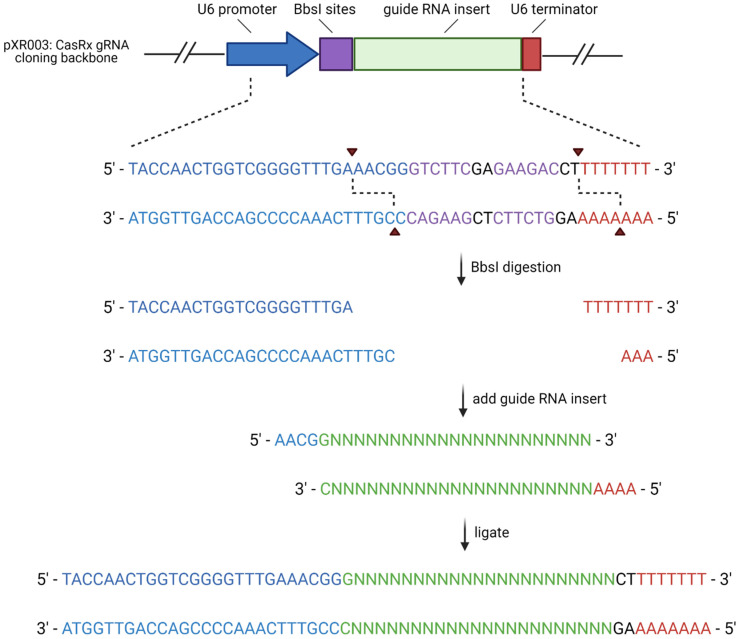
gRNA plasmid cloning workflow. Created in BioRender.com, accessed on 27 September 2021.

A pair of oligonucleotides with a 5′—AACG overhang and a 3′—AAAA overhang were synthesized by Integrated DNA Technologies (IDT) for each of the target sites (Table 1). Each complementary oligo pair was phosphorylated and annealed together by combining 100 μM of each oligo pair with T4 polynucleotide kinase (PNK) and T4 ligation buffer then incubating them in a thermocycler at 37 °C for 30 min, 95 °C for 5 min, and ramping down to 25 °C at 5 °C /min. The oligos were then cloned into the plasmid pXR003. pXR003: CasRx gRNA cloning backbone was a gift from Patrick Hsu (Addgene plasmid #109053; http://n2t.net/addgene:109053, accessed on 23 March 2021; RRID:Addgene_109053) [3]. The plasmid was digested with BbsI restriction enzyme sites and the oligo pairs were ligated into the plasmid with T7 ligase using a thermocycler for 6 cycles at 37 °C for 5 min and 21 °C for 5 min. The plasmid was then treated with PlasmidSafe exonuclease to digest any residual linearized DNA. The cloned plasmids were transformed into Stbl3 cells, and the DNA was purified using a GeneJET Plasmid Miniprep Kit (Thermo Scientific, Waltham, MA, USA, Catalog #K0503). Cloning was verified via Sanger sequencing performed by Eurofins Genomics using a sequencing primer for the hU6 promoter present in pRX003 (5′—GACTATCATATGCTTACCGT—3′). The workflow for this cloning procedure is summarized in Figure 2.

### 2.4. Transfection of Vero Cells

Vero cells were transfected simultaneously with a plasmid encoding CasRx (pCasRx-GFP) and a plasmid encoding guide RNA (pgRNA). Vero cells were seeded on a 24-well plate containing 5 × 10^4^ cells per well and incubated overnight until 70–90% confluent. Cells were transfected with 500 ng DNA of each plasmid using a calcium phosphate transfection kit (Invitrogen, Waltham, MA, USA, Catalog #L3000015) according to the manufacturer’s protocol, adjusting the volumes for the smaller volumes of the 24-well plate [13]. Cells were incubated at 37 °C, 5% CO_2_ overnight. The media was changed, and incubation continued at 37 °C for 24 h. Because the CasRx plasmid also encodes GFP, transfected Vero cells were imaged using an Olympus IX83 microscope under GFP fluorescence to determine the transfection efficiency.

### 2.5. Infecting Transfected Vero Cells with HCoV-OC43

Following transfection with CRISPR components, Vero cells were infected with HCoV-OC43 in RPMI medium at a multiplicity of infection (MOI) of 0.01. Infected Vero cells were incubated at 33 °C, 5% CO_2_ for 1.5 h with continuous rocking. The media was aspirated from the cells and replaced with fresh RPMI + 2% horse serum (RPMI-2). Incubation continued for 4 days, and each day post-infection 200 μL supernatant containing virus was collected for RNA extraction (Zymo Research, Irvine, CA, USA, Catalog #1035) and evaluation by RT-qPCR.

### 2.6. RT-qPCR Assays for HCoV-OC43 Targets

**Figure 3:**
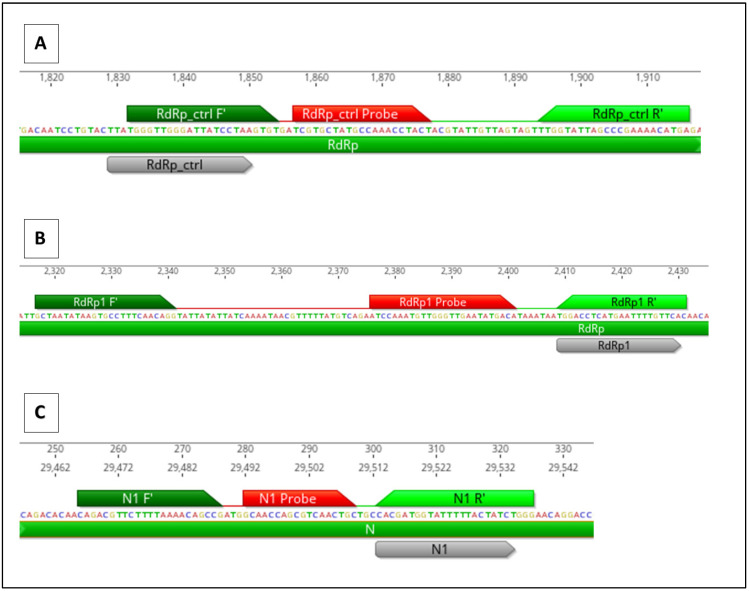
Genomic location of HCoV-OC43 CRISPR gene targets and RT-qPCR assays. CRISPR target sequence is in grey. RT-qPCR forward primers are in dark green, reverse primers are in light green, and probes are in red. (**A**) RdRp_ctrl CRISPR target, (**B**) RdRp CRISPR target, and (**C**) N CRISPR target.

Three RT-qPCR assays were designed that align with each of the CRISPR gene target locations in HCoV-OC43. Primers were constructed to overlap with the specific location of the gRNA target to detect cleavage by CasRx (Figure 3). When CRISPR cleavage occurs at the location of the qPCR primer, the primer is not be able to bind, which results in an increase in the cycle threshold (Ct) value compared to the positive control HCoV-OC43 viral RNA without CRISPR treatment.

Primers and probes were purchased premixed as PrimeTime qPCR assays from IDT. These assays were resuspended to a final stock concentration of 10× using 1000 μL TE buffer. The sequences of the primers and probes used in these assays are in Table 2. 

Each RT-qPCR assay included a final concentration of 1× primer/probe mixed with TaqMan Fast Virus 1-Step Master Mix (Thermo Fisher, Waltham, MA, USA, Catalog #4444434). The reaction was run on a thermocycler at 50 °C for 5 min followed by 95 °C for 20 s, then 40 cycles of 95 °C for 15 s and 60 °C for 1 min. A standard curve was constructed for each of the three RT-qPCR assays to convert the Ct values to HCoV-OC43 genomic equivalents (GE) using the known copy number. The resulting standard curve equations are listed in Table 3 below.

### 2.7. Statistical Analysis

Statistical significance was calculated using one-way ANOVA analyses to determine the differences between the experimental gRNA and the control groups. Statistical significance was defined as a *p* value ≤ 0.05. All statistical analyses were performed in GraphPad Prism software (v 9.3.1, GraphPad Software, LLC, San Diego, CA, USA).

## 3. Results

### 3.1. Selection of Conserved Genes in Human Coronaviruses

RNA viruses such as coronaviruses have a high mutation rate, likely due to their error-prone replication [14]. Certain proteins, such as the spike protein, are affected more by mutations than other genes such as the RdRp gene. Historical data among HCoV-229E and HCoV-OC43 strains have shown that nonsynonymous divergence has gradually increased in the spike protein over the past >30 years while RdRp remains roughly constant and has accrued few if any adaptive substitutions [15]. To develop CRISPR targets that are potentially cross-reactive among coronaviruses, we identified genes that had a high level of homology between coronavirus species and have had minimal adaptive mutations over time. When determining homology, we evaluated individual gene targets rather than comparing whole genome alignment. 

#### 3.1.1. RdRp Gene

RNA-dependent RNA polymerase (RdRp) is a virally encoded gene that is required for genome replication and transcription in RNA viruses such as coronaviruses, making it essential for their survival [16,17,18,19]. This gene has no host cell homolog and plays essential roles in the RNA virus life cycle; therefore, it is critical for RNA viruses to maintain the integrity of this gene. Because of this, there is high conservation of the RdRp gene among viral families, even as new variants and species arise [20]. There is a structurally highly conserved polymerase active site comprising catalytic motifs A-G that are conserved across all RNA viral species [21]. Because the RdRp function is critical for viral survival and it has high conservation among coronaviruses, this gene was chosen as a target to establish pan-coronavirus CRISPR activity. 

#### 3.1.2. N Gene

The nucleocapsid (N) protein also plays an integral role in the replication of viral RNA by packaging its genome inside the viral envelope to aid in the formation of the capsid [5]. The formation of the capsid is needed for viral self-assembly and replication [14,15]. According to previous sequence analysis, the N gene has 34.28–85.41% homology between SARS-CoV-2 and other betacoronaviruses [5]. Like RdRp, the level of homology among HCoV and the critical role this protein has in virus survivability make it a promising target for the CRISPR system. 

### 3.2. Designing gRNA with Mismatches

Based on sequencing alignment and NCBI BLAST analyses, we identified a gRNA sequence in the RdRp gene that is a perfect match between SARS-CoV-2 and HCoV-OC43. We named this gRNA sequence RdRp_ctrl since it acts as a control for exact homology between both viruses. We designed an additional gRNA in a different CRISPR target region of RdRp that contained three nucleotide mismatches between SARS-CoV-2 and HCoV-OC43. To evaluate the effect that mismatches have on CasRx efficiency, we designed additional gRNAs within the same CRISPR target containing between 1 and 3 SNPs. To design these targets with mismatches, nucleotides in the HCoV-OC43 target were substituted for those present in SARS-CoV-2. A similar method was followed to design gRNAs with mismatches for the N gene CRISPR target. Table 4 identifies all of the gRNAs tested, along with the location of the mismatches present. 

### 3.3. Percent Identity of CRISPR Targets to Different HCoV Species

We evaluated our CRISPR targets to determine the percent identity among several HCoV species using the NCBI BLAST database (Table 5). The RdRp_ctrl target had the highest percent identity with 90.9–100% among all HCoV, making it a promising target for a pan-HCoV effector target. 

### 3.4. Transfection Efficiency of pCasRx-GFP

**Figure 4:**
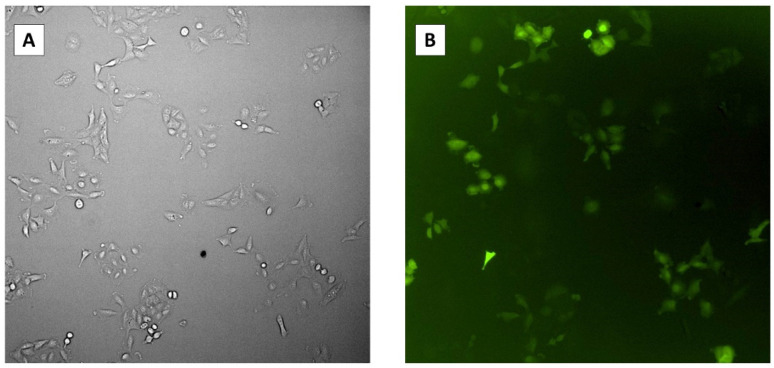
Microscopy images of transfection efficiency 48 h post-transfection. (**A**) Transfected Vero cells under bright field. (**B**) Transfected Vero cells under GFP fluorescence.

We determined the transfection efficiency of the CRIPSR plasmids by detecting green fluorescent protein (GFP) expressed on the pCasRx plasmid inside host cells prior to infection with HCoV-OC43. To achieve this, we transfected Vero cells with the pCasRx-GFP plasmid and determined the presence of GFP inside the cells 48 h post-infection using microscopy (Figure 4). The average percentage of GFP-expressing cells was 60.7% with a standard error of ±1.70%.

### 3.5. Evaluating CasRx Endonuclease Activity in HCoV over Time for Each gRNA Using RT-qPCR

**Figure 5:**
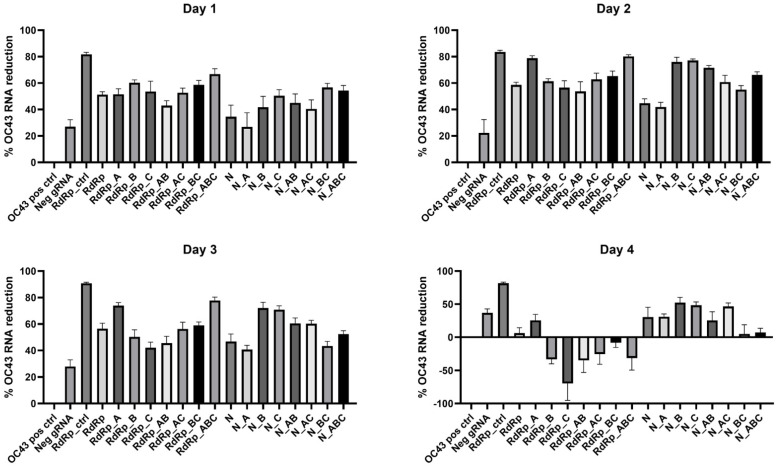
Percent reduction of HCoV-OC43 RNA for each gRNA over time. Data presented as the mean of triplicate replicates with error bars representing the standard error.

We utilized RT-qPCR assays to quantify the CasRx cleavage over time at each specific CRISPR targeting site by designing the assays to overlap with the gRNA region in the gene. We then calculated the percent reduction of HCoV-OC43 RNA present compared to the positive control intact HCoV-OC43 RNA. To appropriately normalize the data, the positive controls were quantified using the same RT-qPCR assays as the gRNA group to which they were being compared. Additionally, we included a negative control assay with a non-targeting gRNA (Neg gRNA) to determine the effects transfection alone had on the production of viral RNA. Because the Neg gRNA did not have a targeting region in HCoV-OC43, we did not design a RT-qPCR assay for this target. Instead, we chose the RdRp_ctrl RT-qPCR assay to quantify OC43 RNA in this group. The results are presented in Figure 5, and the statistical significance of each gRNA compared to both the negative target and positive control are summarized in Table 6 (for more details, see Supplementary File). We found that the overall cleavage efficiency was greatest at two days post-infection. At this timepoint, all gRNA except N_A (*p* = 0.0808) were significantly different than the Neg gRNA. 

### 3.6. Effect of gRNA Mismatches on CasRx Endonuclease Efficacy

Both the number of SNPs and the location of the SNP were evaluated for significance in the RdRp and N CRISPR target locations. 

**Figure 6:**
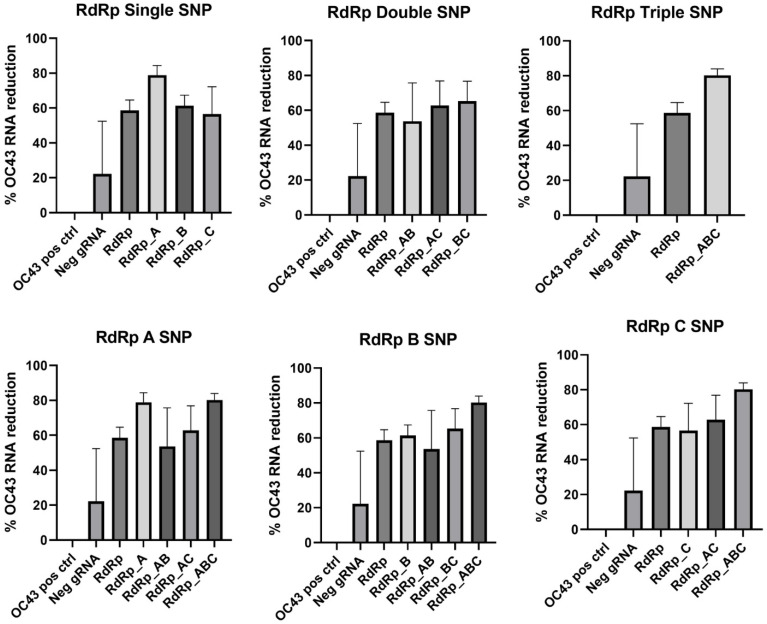
RdRp gRNA mismatch comparison. Data presented as the mean of triplicate replicates with error bars representing the standard error. Graphs in the top row compare number of SNPs while graphs on the bottom compare the SNP location at Day 2 post-infection.

In the RdRp gene target, single SNP at the A location and the RdRp_ABC triple SNP improved the viral RNA reduction the most compared to the RdRp gRNA with no mismatches present, as shown in Figure 6. However, none of the gRNAs containing SNPs were statistically different from the control RdRp gRNA except RdRp_ABC (*p* = 0.0278). *p* values comparing all mismatched RdRp gRNA with the RdRp gRNA with no SNPs are summarized in Table 7.

**Figure 7:**
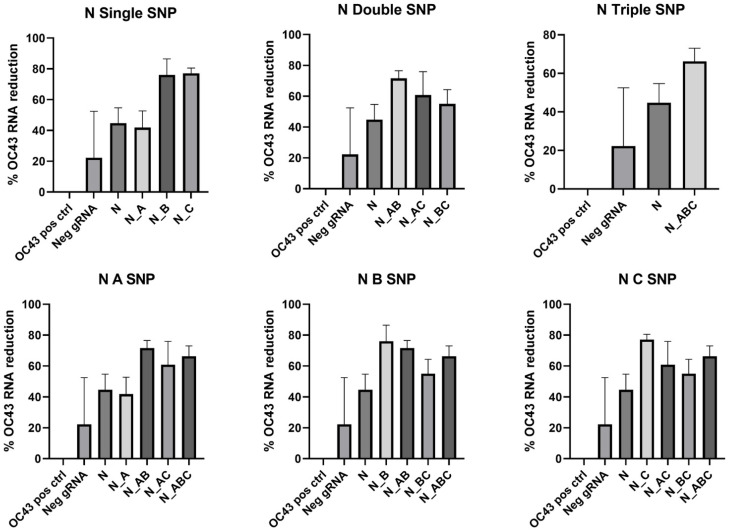
N gRNA mismatch comparison. Data presented as the mean of triplicate replicates with error bars representing the standard error. Graphs in the top row compare number of SNPs while graphs on the bottom compare the SNP location at Day 2 post-infection.

In the N gene target, mismatches in the gRNA sequence improved viral RNA reduction in all cases except for N_A gRNA, in which the mutation decreased the percent reduction in viral RNA. In this CRISPR target location, there was a significantly greater reduction in HCoV-OC43 RNA in N_B, N_C, N_AB, and N_ABC compared to the N gRNA without mismatches (Figure 7, Table 8).

## 4. Discussion

### 4.1. Evaluating the Effect of gRNA SNPs on CasRx Activity

When up to three SNPs are present in a gRNA sequence, CasRx efficacy in reducing viral RNA is equivalent to or improved compared to the gRNA that is an exact match to its viral target. To further examine this, we evaluated the effects of both the location of the mismatch within the guide as well as the number of mismatches present for the RdRp and N gene targets. Previous work has shown that single mismatches can modestly reduce knockdown, but gRNA spacer nucleotides in positions 15–21 are largely intolerant to target site mismatches [6,7]. 

The specific location of the SNP in the gRNA sequence had less impact on the RdRp gRNA than that of the N gRNA. In the N CRISPR target, the N_A gRNA was a disadvantageous mutation while N_B and N_C improved the viral cleavage activity of CasRx. The nucleotide substitution in N_B replaced uracil with cytosine, which disrupted a series of five uracil nucleotides in this guide sequence. This indicates that mismatches may allow otherwise unfavorable gRNA sequences to be adapted for use with CasRx. 

In addition to the location of SNPs, the number of SNPs within a gRNA also had an influence on CasRx activity. There were varying effects when one SNP was present in the gRNA, depending on the guide; some individual SNPs, such as RdRp_A, improved CasRx activity, while others, such as N_A, hindered it. When there were two SNPs in the gRNA, all guides, except N_AB (*p* = 0.0010), were not significantly different from the gRNA with no SNPs. Although we predicted the gRNAs with three SNPs would have a negative effect on CasRx cleavage, we found that these guides significantly improved its nuclease activity in both RdRp and N compared to the guides without mismatches (*p* = 0.0278 and *p* = 0.0304, respectively). Recent work using an energy-based model with Cas9 has found that interactions between gRNA and the targeted DNA may influence cleavage activity, resulting in Cas9 cleaving off-target sites with higher efficiency than on-target sites [22,23]. It is possible there is a similar interaction between gRNAs and targeted RNA when Cas13 is used, but this should be further elucidated in future work using a robust number of gRNA sequences.

We found that not only are mutations in the gRNA important, but the location of the CRISPR target within the gene also seems to impact efficacy. This is evidenced by comparing RdRp_ctrl and RdRp gRNAs, which were located in different regions of the RdRp gene. RdRp_ctrl had a significantly better efficiency than seen in the RdRp target (*p* = 0.0034). This may be in part due to a tertiary structure that hinders the ability of CasRx to access specific regions of the targeted gene. This supports previous work that has found there are position-dependent effects when targeting different regions of a CRISPR target region [6,8]. There are numerous computational algorithms that can predict different levels of protein structure to facilitate the design of gRNAs in target regions that are more accessible to the Cas effector [24].

### 4.2. Other Factors Affecting CasRx Activity

#### 4.2.1. Transfection Affects the Efficacy of Viral Replication

Transfection of host cells with CasRx and non-targeting Neg gRNA reduced HCoV-OC43 RNA up to 28.0% by Day 3 post-infection. This may be due to that fact that host cell machinery is required to transcribe plasmid DNA, leaving less cellular resources available for the virus to use to replicate itself upon infection. In previous work, it has also been shown that active CasRx may cause mammalian cells to die or have a growth disadvantage [25]. Additionally, other reports have indicated that CasRx may be toxic in Drosophila, human U87 glioblastoma cells, and mouse embryonic stem cells [26,27]. This cell toxicity may affect the ability for host cells to propagate HCoV-OC43 in the presence of CasRx, thereby contributing to the reduction of viral RNA observed. 

#### 4.2.2. Temporal Changes

The greatest percent reduction in HCoV-OC43 viral RNA was observed at Day 2 post-infection. By Day 4 post-infection, only the RdRp_ctrl gRNA reduced viral RNA significantly compared to the positive control sample (*p* = 0.0003). This suggests that CasRx CRISPR cleavage delays the progression of an HCoV-OC43 infection in host cells but does not prevent it. This may be a result of the transfection efficiency of the Vero cells with CRISPR components. Because transfection with CRISPR plasmids was less than 100%, viruses are able to infect and replicate in host cells lacking CasRx or gRNAs. By Day 4, this resulted in higher HCoV-OC43 concentrations, even in samples with previously effective CRISPR targets.

## 5. Conclusions

We have shown that SNPs in the gRNA sequence can affect the efficacy of CasRx, and this CRISPR effector can tolerate up to three mismatches in the gRNA sequence while maintaining its nuclease activity. This allows for less sequence specificity to be targeted with a single gRNA using CasRx, which is important in establishing a pan-HCoV effector system where perfect sequence identity is not present among all species. 

While there has been work on establishing off-target effects in Cas9 [28,29], not all Cas enzymes behave similarly with regard to off-target effects, as demonstrated by our current work. Because our study evaluated the effects of SNPs in only two viral genes, there is much more work to be done in this area to further elucidate the effects of mismatches in gRNA sequences more broadly. To make our findings more broadly extensible, it is also necessary to determine the maximum number of SNPs that can be present for CasRx to remain effective. It is evident that sequence optimization of gRNA is an important aspect to optimizing nuclease efficiency when utilizing CasRx. The degree of off-target effects needs to be strictly evaluated for safety when using CasRx. 

Because CasRx appears to be tolerant of certain SNPs within gRNA sequences, it is possible that guides can cross-react with similar species among the same viral family, especially in highly conserved regions such as the RdRp gene. This would allow CRISPR targets to be preliminarily characterized in surrogate organisms without having to handle more pathogenic species. Additionally, there would not need to be as much homology between the model organism and the virulent one when targets are selected. This increases confidence when translating work from model organisms to more pathogenic species. As such, the gRNAs tested here may be viable targets in other coronaviruses such as SARS-CoV-2. 

Furthermore, because CasRx is tolerant of mismatches, CRISPR guides can target pathogens more broadly at the family level by targeting conserved essential genes. This approach allows targeting of newly evolved variants or even new pathogenic species within a family, thereby giving us persistent tools against future pandemics and emerging threats.

## Figures and Tables

**Table 1:** Oligonucleotide sequences of gRNA cloned into pXR003. Colored nucleotides correlate to the colors in Figure 2 to designate the promotor, gRNA insert, and terminator sequences.

gRNA Name *	Top Strand (5′ to 3′)	Bottom Strand (5′ to 3′)
RdRp_ctrl	AACGGTTATGGGTTGGGATTATCCTAA	AAAATTAGGATAATCCCAACCCATAAC
RdRp	AACGGTGGACCTCATGAATTTTGTTCA	AAAATGAACAAAATTCATGAGGTCCAC
RdRp_A	AACGGAGGACCTCATGAATTTTGTTCA	AAAATGAACAAAATTCATGAGGTCCTC
RdRp_B	AACGGTGGACCTCATGAATTTTGCTCA	AAAATGAGCAAAATTCATGAGGTCCAC
RdRp_C	AACGGTGGACCTCATGAATTTTGTTCT	AAAAAGAACAAAATTCATGAGGTCCAC
RdRp_AB	AACGGAGGACCTCATGAATTTTGCTCA	AAAATGAGCAAAATTCATGAGGTCCTC
RdRp_AC	AACGGAGGACCTCATGAATTTTGTTCT	AAAAAGAACAAAATTCATGAGGTCCTC
RdRp_BC	AACGGTGGACCTCATGAATTTTGCTCT	AAAAAGAGCAAAATTCATGAGGTCCAC
RdRp_ABC	AACGGAGGACCTCATGAATTTTGCTCT	AAAAAGAGCAAAATTCATGAGGTCCTC
N	AACGGCACGATGGTATTTTTACTATCT	AAAAAGATAGTAAAAATACCATCGTGC
N_A	AACGGCAAGATGGTATTTTTACTATCT	AAAAAGATAGTAAAAATACCATCTTGC
N_B	AACGGCACGATGGTATTTCTACTATCT	AAAAAGATAGTAGAAATACCATCGTGC
N_C	AACGGCACGATGGTATTTTTACTACCT	AAAAAGGTAGTAAAAATACCATCGTGC
N_AB	AACGGCAAGATGGTATTTCTACTATCT	AAAAAGATAGTAGAAATACCATCTTGC
N_AC	AACGGCAAGATGGTATTTTTACTACCT	AAAAAGGTAGTAAAAATACCATCTTGC
N_BC	AACGGCACGATGGTATTTCTACTACCT	AAAAAGGTAGTAGAAATACCATCGTGC
N_ABC	AACGGCAAGATGGTATTTCTACTACCT	AAAAAGGTAGTAGAAATACCATCTTGC
Neg gRNA	AACGGATCTATTGTTCCGACGTATTAT	AAAATAGATAACAAGGCTGCATAATAC

* A, B, and C in gRNA name represent location of SNPs in gRNA.

**Table 2:** RT-qPCR oligo sequences. 6FAM = 6-carboxyfluorescein; MGBNFQ = minor groove binder non-fluorescent quencher.

Target	Oligo Name	Sequence (5′ → 3′)	T_m_ (°C)
RdRp_ctrl	Forward primer	TGGGTTGGGATTATCCTAAGTGT	58.9
	Probe	6FAM—TCGTGCTATGCCAAACCTACT—MGBNFQ	59.4
	Reverse primer	TCATGTTTTCGGGCTAATACCAA	58.4
RdRp	Forward primer	GCTAATATAAGTGCCTTTCAACAGG	58.3
	Probe	6FAM—ATCCAAATGTTGGGTTGAATATGACA—MGBNFQ	59.6
	Reverse primer	GTGAACAAAATTCATGAGGTCCA	57.5
N	Forward primer	CAGACGTTCTTTTAAAACAGCCG	59.0
	Probe	6FAM—GCAACCAGCGTCAACTGC—MGBNFQ	60.1
	Reverse primer	CCCAGATAGTAAAAATACCATCGTG	57.6

**Table 3:** RT-qPCR standard curve equations to convert Ct value to genomic equivalents (GE).

RT-qPCR Target	Standard Curve Equation
RdRp_ctrl	$\mathrm{GE}=10^{\frac{\mathrm{Ct}-55.911}{-3.4999}}$
RdRp	$\mathrm{GE}=10^{\frac{\mathrm{Ct}-53.351}{-3.3443}}$
N	$\mathrm{GE}=10^{\frac{\mathrm{Ct}-56.727}{-3.4599}}$

**Table 4:** gRNA sequences and mismatch location. A, B, and C in gRNA name represent SNPs in the gRNA sequence. Orange nucleotides match SARS-CoV-2 sequence; blue nucleotides match OC43 sequence; black nucleotides are consensus sequences.

CRISPR Target	gRNA Name	Targeting Sequence (5′ → 3′)	# of OC43 Mismatches	# of SARS-CoV-2 Mismatches	Notes
RdRp_ctrl target	RdRp_ctrl	UUAUGGGUUGGGAUUAUCCUAA	0	0	Target is 100% homologous between OC43 and SARS-CoV-2
RdRp target	RdRp	**U**GGACCUCAUGAAUUUUG**U**UC**A**	0	3	Target has 3 mismatches between OC43 and SARS-CoV-2 at nucleotide positions 1 [A], 19 [B], and 22 [C]
	RdRp_A	**A**GGACCUCAUGAAUUUUG**U**UC**A**	1	2	
	RdRp_B	**U**GGACCUCAUGAAUUUUGCUC**A**	1	2	
	RdRp_C	**U**GGACCUCAUGAAUUUUG**U**UC**U**	1	2	
	RdRp_AB	**A**GGACCUCAUGAAUUUUGCUC**A**	2	1	
	RdRp_AC	**A**GGACCUCAUGAAUUUUG**U**UC**U**	2	1	
	RdRp_BC	**U**GGACCUCAUGAAUUUUGCUC**U**	2	1	
	RdRp_ABC	**A**GGACCUCAUGAAUUUUGCUC**U**	3	0	
N target	N	CA**C**GAUGGUAUUU**U**UACUA**U**CU	0	3	Target has 3 mismatches between OC43 and SARS-CoV-2 at nucleotide positions 3 [A], 14 [B], and 20 [C]
	N_A	CA**A**GAUGGUAUUU**U**UACUA**U**CU	1	2	
	N_B	CA**C**GAUGGUAUUU**C**UACUA**U**CU	1	2	
	N_C	CA**C**GAUGGUAUUU**U**UACUA**C**CU	1	2	
	N_AB	CA**A**GAUGGUAUUU**C**UACUA**U**CU	2	1	
	N_AC	CA**A**GAUGGUAUUU**U**UACUA**C**CU	2	1	
	N_BC	CA**C**GAUGGUAUUU**C**UACUA**C**CU	2	1	
	N_ABC	CA**A**GAUGGUAUUU**C**UACUA**C**CU	3	0	

**Table 5:** Percent identity of CRISPR targets with HCoV species.

CRISPR Target	MERS-CoV	SARS-CoV	SARS-CoV-2	HCoV-OC43	HCoV-2293	HCoV-HKU1
RdRp_ctrl target	100%	100%	100%	100%	90.9%	100%
RdRp target	90.9%	77.3%	86.4%	100%	77.3%	100%
N target	63.6%	63.6%	95.5%	100%	63.6%	81.8%

**Table 6:** One-way ANOVA comparison of gRNA with positive control and negative gRNA. *p* values with an asterisk indicate the percent reduction is not significantly different from that of the HCoV-OC43 control or negative gRNA.

CRISPR Target	Day 1		Day 2		Day 3		Day 4	
	ANOVA with Pos Ctrl	ANOVA with Neg gRNA	ANOVA with Pos Ctrl	ANOVA with Neg gRNA	ANOVA with Pos Ctrl	ANOVA with Neg gRNA	ANOVA with Pos Ctrl	ANOVA with Neg gRNA
RdRp_ctrl	<0.0001	<0.0001	<0.0001	<0.0001	<0.0001	<0.0001	0.0003	* 0.4057
RdRp	<0.0001	* 0.1637	<0.0001	<0.0001	<0.0001	<0.0001	* >0.9999	* 0.9342
RdRp_A	<0.0001	* 0.1532	<0.0001	<0.0001	<0.0001	<0.0001	* 0.9891	* >0.9999
RdRp_B	<0.0001	0.0041	<0.0001	<0.0001	<0.0001	0.0077	* 0.8785	0.0052
RdRp_C	<0.0001	* 0.0728	<0.0001	<0.0001	<0.0001	* 0.4619	0.0058	<0.0001
RdRp_AB	<0.0001	* 0.8441	<0.0001	<0.0001	<0.0001	* 0.1203	* 0.8285	0.0036
RdRp_AC	<0.0001	* 0.1010	<0.0001	<0.0001	<0.0001	<0.0001	* 0.9896	0.0270
RdRp_BC	<0.0001	0.0089	<0.0001	<0.0001	<0.0001	<0.0001	* >0.9999	* 0.3854
RdRp_ABC	<0.0001	0.0001	<0.0001	<0.0001	<0.0001	<0.0001	* 0.9232	0.0079
N	0.0022	* >0.9999	<0.0001	0.0169	<0.0001	* 0.0656	* 0.9374	* >0.9999
N_A	* 0.0689	* >0.9999	<0.0001	* 0.0808	<0.0001	* 0.6632	* 0.9275	* >0.9999
N_B	<0.0001	* 0.9173	<0.0001	<0.0001	<0.0001	<0.0001	* 0.1521	* >0.9999
N_C	<0.0001	* 0.2103	<0.0001	<0.0001	<0.0001	<0.0001	* 0.2655	* >0.9999
N_AB	<0.0001	* 0.6830	<0.0001	<0.0001	<0.0001	<0.0001	* 0.9895	* >0.9999
N_AC	<0.0001	* 0.9632	<0.0001	<0.0001	<0.0001	<0.0001	* 0.3332	* >0.9999
N_BC	<0.0001	0.0214	<0.0001	<0.0001	<0.0001	* 0.3055	* >0.9999	* 0.9102
N_ABC	<0.0001	0.0560	<0.0001	<0.0001	<0.0001	0.0018	* >0.9999	* 0.9514
Neg gRNA	* 0.0663	-	0.0190	-	<0.0001	-	* 0.7501	-

**Table 7:** One-way ANOVA comparison of RdRp gRNA mismatches. *p* values with an asterisk indicate the percent reduction is not significantly different from that of the RdRp control gRNA.

CRISPR Target	ANOVA with RdRp
RdRp_A	* 0.0576
RdRp_B	* >0.9999
RdRp_C	* >0.9999
RdRp_AB	* >0.9999
RdRp_AC	* >0.9999
RdRp_BC	* 0.9996
RdRp_ABC	0.0278

**Table 8:** One-way ANOVA comparison of N gRNA mismatches. *p* values with an asterisk indicate the percent reduction is not significantly different from that of the N control gRNA.

CRISPR Target	ANOVA with N
N_A	* >0.9999
N_B	<0.0001
N_C	<0.0001
N_AB	0.0010
N_AC	* 0.3509
N_BC	* 0.9542
N_ABC	0.0304

## Data Availability

The datasets generated during and analyzed during the current study are available from the corresponding author upon reasonable request.

## Supplementary Materials

The following supporting information can be downloaded at https://www.mdpi.com/article/10.3390/cells11121859/s1, Raw data of the experiments.
